# Growing up after childhood cancer: maturity and life satisfaction in young adulthood

**DOI:** 10.1007/s00520-021-06260-3

**Published:** 2021-05-07

**Authors:** Taylor M. Dattilo, Randal S. Olshefski, Leena Nahata, Jennifer A. Hansen-Moore, Cynthia A. Gerhardt, Vicky Lehmann

**Affiliations:** 1 Center for Biobehavioral Health, The Abigail Wexner Research Institute At Nationwide Children’s Hospital, Columbus, OH USA; 2 Division of Hematology/Oncology/BMT, Nationwide Children’s Hospital, Columbus, OH USA; 3 Divison of Endocrinology, Nationwide Children’s Hospital, Columbus, OH USA; 4 Department of Pediatrics, The Ohio State University College of Medicine, Columbus, OH USA; 5 Division of Pediatric Psychology and Neuropsychology, Nationwide Children’s Hospital, Columbus, OH USA; 6 Department of Medical Psychology, Amsterdam University Medical Centers, Cancer Center Amsterdam, University of Amsterdam, Meibergdreef 9, 1105AZ Amsterdam, The Netherlands

**Keywords:** Childhood cancer, Life satisfaction, Maturity, Oncology, Survivor, Young adulthood

## Abstract

**Purpose:**

Young individuals face a variety of developmental tasks as they mature into adulthood. For survivors of childhood cancer, growing up may be more difficult due to their illness and late effects from treatment. This study is the first to quantitatively examine perceptions of maturity and how these perceptions contribute to satisfaction with life among young adult survivors of childhood cancer.

**Methods:**

Ninety survivors of childhood cancer (*M*_age_ = 29.8; 7–37 years post-diagnosis) were recruited to complete online surveys on how mature they felt relative to peers, their perceived maturity on three domains (financial, personal, social), and life satisfaction.

**Results:**

Most survivors (62%; *n* = 56) felt they grew up faster than their peers, and over half (56%; *n* = 50) felt more mature. Perceived maturity was high on all three domains, but brain tumor survivors reported significantly lower maturity than other survivors (*d* = 0.76–1.11). All maturity domains were positively associated with life satisfaction (*r* = .49–.56). Hierarchical linear regressions indicated that 44% of the variance in life satisfaction was explained by perceptions of growing up slower (*β* =  − 1.08, *p* = .004) and marginally by greater perceived personal maturity (*β* = 0.45, *p* = .061).

**Conclusions:**

Childhood cancer can influence development, with most survivors feeling that they grew up faster and were more mature than peers. Personal maturity was related to life satisfaction, with survivors of brain tumors or those who felt they grew up slower at greatest risk for lower life satisfaction. Future research and clinical practice should consider survivors’ development and maturation across the life span to promote overall well-being.

## Background

Survival rates for childhood cancer now exceed 80% [1, 2]. As a result, most survivors will grow up and transition into new life phases, such as emerging and young adulthood [2]. These phases are marked by significant change and growth that largely define them as the adults that they will become. Key developmental tasks during this time focus on future ambitions and goals, like choosing a career, living independently, or starting a family [3, 4]. Achievement of these socially valued milestones often builds on one another and is, therefore, an important step toward maturity and adulthood. While such transitions may be exciting, they can also be challenging, especially for childhood cancer survivors [5]. Cancer treatment itself and the emergence of late effects can disrupt physical and psychosocial development [5, 6]. Yet, limited research has examined how cancer affects perceptions of maturity in young adult survivors of childhood cancer.

Qualitative research among survivors of childhood cancer indicates a “dual reality,” where developmental changes can be experienced as both positive and negative [7–10]. For example, young adult survivors can experience positive emotions and have an optimistic attitude towards life after overcoming cancer, while also recognizing the continued challenges of survivorship [7]. Survivors report growing up faster and feeling more mature than peers [8, 11], but also note the social cost of early maturation, including the loss of childhood innocence and normalcy, missed opportunities, and feeling different/alienated from peers [9, 12, 13]. In contrast, some survivors, particularly those who had brain tumors or neurotoxic therapies, may delay or fail to achieve certain developmental milestones [14, 15]. For example, neurocognitive impairments may negatively affect educational attainment or contribute to social difficulties [16–18]. These challenges can further cascade into problems navigating social interactions, such as dating and romantic/sexual relationships [6, 19–21]. Importantly, milestones of social maturity, such as having meaningful friendships, engaging in romantic relationships, or getting married, are key indicators used by many people to rate their success and happiness in life [22]. As a result, some survivors may be at risk for diminished well-being and lower satisfaction with life in adulthood [23]. Yet, whether and to what extent different aspects of maturation play a role in survivors’ overall life satisfaction remains unknown.

Some studies among adult survivors of childhood cancer reported compromised satisfaction with life [24, 25], whereas others found no differences relative to the general population [26–28]. Moreover, little is known about factors that contribute to this variability in life satisfaction. For example, potential sex differences remain largely unknown as there are mixed findings between male and female survivors [24–28]. Additionally, two studies have indicated lower life satisfaction among brain tumor survivors [23, 27]. Thus, it is important to understand how childhood cancer affects perceptions of development or maturation, and whether these perceptions contribute to life satisfaction.

Therefore, we examined associations between the perceived pace of growing up, maturity, and life satisfaction among young adult survivors of childhood cancer. It was hypothesized that most survivors report growing up faster and feeling more mature than peers, and we expected a positive association of maturity and life satisfaction. Given limited previous evidence, we also explored the effects of background factors on maturity and satisfaction with life.

## Methods

### Study design

Data were part of a larger study on the psychosexual development of childhood cancer survivors [21, 29]. Eligible survivors were 20–40 years old, diagnosed between ages 5 and 18 years at a large pediatric academic hospital in the Midwest of the USA, and were ≥ 5 years post-diagnosis. They were initially recruited in 2013–2015, resulting in 149 participants. In 2016, participating survivors were invited to complete a follow-up assessment. The Institutional Review Board (IRB16-00426) approved all procedures.

Of the 149 survivors eligible for follow-up, 2 had died, and at least 28 had outdated contact information. Therefore, 119 (80%) survivors potentially received the invitation letter, and 92 (62%) visited the study website and participated. Written informed consent was provided online before survivors began the survey. Two survivors had incomplete data and were excluded from the presented analyses, resulting in a final sample of *N* = 90 survivors. Participants were 29.82 years old (SD = 5.12; range: 22–43 years), had been diagnosed an average age of 11.84 (*SD* = 3.61, range: 5–18), and were currently 7–37 years (*M* = 17.98, SD = 5.63) post-diagnosis (see additional information in Table 1).

**Table 1:** Demographic characteristics and outcomes of perceived maturity and satisfaction with life (SwL)

	*n(%)*	Maturity	Financial maturity	Personal maturity	Social maturity	SwL
		*M* (SD)	*M* (SD)	*M* (SD)	*M* (SD)	*M* (SD)
Total sample	90 (100%)	4.1 (0.8)	4.2 (0.9)	4.1 (0.8)	4.1 (0.9)	5.4 (1.3)
Sex						
Female	56 (62.2%)	4.1 (0.8)	4.1 (1.1)	4.1 (0.7)	4.0 (1.0)	5.4 (1.4)
Male	34 (37.8%)	4.3 (1.2)	4.4 (0.7)	4.2 (0.7)	4.2 (0.8)	5.5 (1.2)
Relationship status						
Single	24 (26.7%)	**3.7 (0.8)**	3.9 (1.0)	3.9 (0.7)	**3.3 (1.0)**	**4.8 (1.4)**
Partnered	66 (73.3%)	**4.3 (0.7)**	4.3 (0.9)	4.2 (0.8)	**4.4 (0.8)**	**5.7 (1.2)**
Level of education						
High	62 (68.9%)	**4.4 (0.6)**	**4.5 (0.7)**	**4.4 (0.7)**	**4.4 (0.7)**	**5.7 (1.1)**
Low	28 (31.1%)	**3.6 (0.8)**	**3.5 (1.0)**	**3.7 (0.8)**	**3.5 (1.1)**	**4.8 (1.4)**
Cancer diagnosis						
Leukemia	26 (28.9%)	4.2 (0.6)	4.3 (0.8)	4.2 (0.6)	4.2 (0.9)	5.4 (1.2)
Brain tumor	23 (25.6%)	**3.6 (0.9)**^**a**^	3.7 (1.2)	**3.7 (0.9)**^**b**^	**3.4 (1.2)**^**a**^	**4.9 (1.4)**^**c**^
Lymphoma	22 (24.4%)	4.4 (0.7)	4.3 (0.9)	4.4 (0.7)	4.4 (0.6)	5.5 (1.4)
Other solid tumor	19 (21.1%)	4.4 (0.6)	4.3 (0.8)	4.4 (0.5)	4.4 (0.7)	6.1 (0.6)
Neurotoxic treatment						
None	29 (32.2%)	4.3 (0.7)	4.3 (0.9)	4.3 (0.7)	4.3 (0.8)	5.8 (0.9)
Low dose	22 (24.4%)	4.2 (0.6)	4.4 (0.7)	4.1 (0.8)	4.1 (0.9)	5.3 (1.3)
High dose	39 (43.3%)	4.0 (0.9)	4.0 (1.1)	4.1 (0.8)	3.9 (1.1)	5.2 (1.5)
Age at diagnosis						
≤ age 12	49 (54%)	4.0 (0.8)	4.0 (1.1)	4.0 (0.8)	4.0 (1.0)	5.2 (1.4)
> age 12	41 (46%)	4.3 (0.7)	4.4 (0.8)	4.2 (0.7)	4.3 (0.8)	5.7 (1.1)

Significant differences are bolded

^**a**^Differed significantly from all other subgroups

^**b**^Differed significantly from lymphoma and other solid tumor survivors

^**c**^Differed significantly from other solid tumor survivors

### Measures

#### Demographic and medical characteristics

Participants self-reported basic demographic information online, including age, sex, relationship status, and level of education. Research staff completed medical chart reviews to collect medical characteristics, including age at diagnosis, type of diagnosis, and extent of neurotoxic treatment intensity (none, low, high [21]).

#### Perceived maturity

Based on the Adult Identity Profiles [30], two items were used to assess survivors’ perceived growth and maturity relative to their own peers: “How fast did you grow up with regard to your same aged peers?” (response categories: “faster,” “about the same,” and “slower”), and “Compared to your peers, do you feel more or less mature?” (response categories: “I feel more mature than my peers,” “I feel we are all quite similar,” and “I feel less mature than my peers”).

Due to a lack of standardized measures on self-perceived maturity, the study team created a 10-item Maturity Questionnaire (Table 2), which included a total score and three subscales: financial maturity, personal maturity, and social maturity. The *financial* maturity subscale includes three items assessing how adult/mature survivors felt about their financial independence, school/job competency, and managing their own responsibilities (e.g., bills, insurance). The *personal* maturity subscale includes four items: emotional stability, autonomy, managing their own health, and ambitions/goals for the future. The *social* maturity subscale includes three items: assessing friendships/social relationships, dating/romantic relationships, and family planning. All items were answered on a 5-point scale: “not at all”– “very adult/mature.” Mean scores were calculated with higher scores indicating greater perceived maturity. Internal consistency was excellent for the total score (*α* = .90) and very good for the three subscales: financial maturity (*α* = .83), personal maturity (*α* = .81), and social maturity (*α* = .83).

**Table 2:** Items of the Maturity Questionnaire as ranked by survivors

		*M* (SD)	Rank by *M*	Endorsed “very mature” %	Rank by %
F	School/job competence	4.3 (0.9)	1	55.6%	1
P	Managing own health	4.3 (0.8)	2	45.6%	6
F	Managing own responsibilities (e.g., bills, insurances)	4.2 (1.0)	3	52.2%	2
P	Autonomy	4.2 (0.9)	4	47.8%	5
S	Friendships/social relationships	4.2 (1.0)	5	44.4%	7
P	Ambitions/goals for the future	4.1 (1.0)	6	44.4%	7
S	Dating/romantic relationships	4.1 (1.2)	7	50.0%	3
S	Family planning	4.0 (1.1)	8	44.4%	7
P	Emotional stability	4.0 (1.0)	9	34.4%	8
F	Financial independence	3.9 (1.3)	10	49.9%	4

*F *Financial maturity, *S* Social maturity, *P* Personal maturity

#### Satisfaction with life

The Satisfaction with Life Scale (SWLS [31]) is a widely used 5-item questionnaire to assess the degree to which participants are satisfied with life. Items are rated on a 7-point scale (“strongly disagree”– “strongly agree”) and include statements such as “In most ways my life is close to ideal.” Higher scores indicate greater satisfaction and internal consistency was excellent (*α* = .91).

### Statistical analyses

Data were analyzed using SPSS Statistics by IBM, version 25.0. Descriptive statistics will be used to summarize survey responses. Differences in maturity based on demographic and medical factors will be examined using *t*-tests, one-way ANOVAs, or Pearson’s correlation, depending on the variable: sex, age, relationship status (partnered vs. single), educational level (low vs. high), age at diagnosis (childhood vs. adolescence), type of diagnosis, and extent of neurotoxic treatment regimens (no, low, high [21]). To test the relative contribution of demographic and medical factors, only those significant at *p* < 0.05 will be included in a linear regression analysis on total maturity scores.

Differences in life satisfaction by demographic and medical factors will similarly be examined as indicated above (i.e., using *t*-tests, one-way ANOVAs, or Pearson’s correlation, depending on the variable). Associations between both primary outcomes (i.e., maturity and life satisfaction) will be tested using Pearson’s correlations. Finally, a hierarchical linear regression will test the relative contribution of significant demographic and medical factors and maturity on life satisfaction. Thus, demographic and medical factors that will be identified as significantly related to life satisfaction in univariate analyses (*p* < 0.05) are entered in step 1, followed by the three maturity subscales in step 2.

## Results

### Maturity

Sixty-two percent of survivors (*n* = 56) felt they grew up faster than their peers, while only 12% (*n* = 11) felt they grew up slower. Fifty-six percent (*n* = 50) felt more mature than their peers, while 11% (*n* = 10) felt less mature. Of all 10 items included in the Maturity Questionnaire, the highest mean scores were reported for school/job competency and managing one’s health, whereas lowest scores were reported for emotional stability and financial independence (Table 2). However, when examining these items by how many times survivors endorsed the highest possible response category (“very mature”), a slightly different distribution appeared. Both distributions showed high perceived maturity on school/job competency and managing one’s own responsibilities, but the least frequently endorsed items included family planning, ambitions/goals, and friendships/social relationships (see Table 2 for direct comparisons).

The total score for perceived maturity was high (*M* = 4.1), given a potential range of 1–5, and similar across subscales: financial (*M* = 4.2), personal (*M* = 4.1), and social maturity (*M* = 4.0, Table 2). Testing whether demographic and medical factors were related to maturity showed that partnered survivors perceived themselves as more mature than single survivors (*M* = 4.3 vs. 3.7), *t*(88) =  − 3.13, *p* = 0.002; *d* = 0.72, particularly for *social* maturity (*M* = 4.4 vs. 3.3), *t*(88) =  − 4.66, *p < *.001, *d* = 1.18 (Table 1). Moreover, older age at study participation was weakly associated with greater maturity, *r* = 0.22, *p* = 0.038. Importantly, partnered survivors were older than singles (*M* = 28.0 vs. 30.5), *t*(88) =  − 2.12, *p* = 0.037, but testing maturity differences among partnered and single survivors, while controlling for age, did not change the findings reported above. Finally, higher educated survivors perceived themselves as more mature than those with a lower education (*M* = 4.4 vs. 3.6), *t*(88) = 5.45, *p* < 0.001; *d* = 1.18, while sex was not related to perceived maturity.

Regarding medical factors, total maturity scores differed by the type of diagnosis, *F*(3,86) = 5.45, *p* = 0.002. Brain tumor survivors (*M* = 3.6) reported significantly lower maturity scores than survivors of leukemia (*M* = 4.2, *d* = 0.76), lymphoma (*M* = 4.4, *d* = 0.90), and other solid tumors (*M* = 4.4, *d* = 1.11). At the subscale level, it appeared that this difference was driven by *social* maturity, where brain tumor survivors reported the lowest scores relative to all other diagnostic groups, *F*(3,86) = 6.26, *p* = 0.001, *d* = 0.25–0.28 (Table 1). Brain tumor survivors also reported significantly lower *personal* maturity (*M* = 3.68) than survivors of lymphoma (*M* = 4.4) and other solid tumors (*M* = 4.4), *F*(3,86) = 4.70, *p* = 0.004, *d* = 0.83–0.99 (Table 1). There was a borderline significant difference regarding age at diagnosis, *F*(1,88) = 3.92, *p* = 0.051. Specifically, those diagnosed during childhood (≤ age 12) reported lower maturity scores compared to survivors diagnosed during adolescence (*M* = 4.0 vs. 4.3, *d* = 0.42). Finally, neurotoxic treatment regimens appeared unrelated to perceived maturity (Table 1).

The overall regression model on maturity was significant, *F*(5,84) = 10.77, *p* < 0.001, explaining 39.1% of the total variance. A brain tumor diagnosis (*β* =  − 0.30, *p* = 0.002) and lower education (*β* =  − 0.46, *p* < 0.001) were associated with lower perceived maturity (Table 3).

**Table 3:** Linear regression on total maturity scores

Variable	Maturity		
	*B* (SE)	*β*	*p*-value
Relationship status: partnered^a^	0.13 (0.17)	0.07	.454
Age at study participation	− 0.01 (0.01)	− 0.03	.746
Education: lower^b^	− 0.75 (0.15)	− 0.46	**.000**
Diagnosis: brain tumor^c^	− 0.53 (0.17)	− 0.30	**.002**
Age at diagnosis: adolescence^d^	0.19 (0.14)	0.12	.173

*R*^2^ = .39, *F*(5,84) = 10.77, *p* < .001; reference categories: ^a^vs. single; ^b^vs. high; ^c^vs. non-CNS; ^d^vs. childhood

### Satisfaction with life

Life satisfaction was high (*M* = 5.4) on a scale from 1–7. Partnered survivors were more satisfied with life than single survivors (*M* = 5.7 vs. 4.8), *t*(88) =  − 2.94, *p* = 0.004, *d* = 0.66, and those with higher education were more satisfied than those with a lower education (*M* = 5.7 vs. 4.8), *t*(88) = 3.35, *p* = 0.001, *d* = 0.73. Moreover, satisfaction differed by type of diagnosis, *F*(3,85) = 3.35, *p* = 0.023. Specifically, brain tumor survivors were significantly less satisfied with life (*M* = 4.9) than survivors of other solid tumors (*M* = 6.1, *d* = 1.12). Sex, age at study, age at diagnosis, and neurotoxic treatment regimens were not associated with life satisfaction (Table 1).

Although subgroups were small, survivors’ pace of growing up appeared to matter for satisfaction with life. Survivors who indicated having grown up slower than peers (*n* = 11) were significantly less satisfied with life (*M* = 3.9) than those who reportedly grew up faster (*n* = 56; *M* = 5.7, *d* = 1.55) and those who felt similar to peers (*n* = 23, *M* = 5.5, *d* = 1.32; *F*(2,87) = 11.72, *p* < 0.001), which are considered very large differences. Most of these survivors were female (72.7%; *n* = 8/11) and/or diagnosed during childhood (63.6%; *n* = 7/11), but subgroups were too small to test differences statistically.

Bivariate correlations between satisfaction with life and the three maturity subscales showed moderate associations. The strongest correlation was between life satisfaction and personal maturity (*r* = 0.56, *p* < 0.001), followed by financial and social maturity (*r* = 0.49, *p* < 0.001 each).

Based on differences identified above, the final hierarchical linear regression included relationship status, education, type of diagnosis, and perceptions of pace of growing up in step 1, before adding the three maturity subscales in step 2. The overall model in step 1 was significant, *F*(4,85) = 10.59, *p* < 0.001, explaining 33.2% of the total variance. Perceptions of growing up slower (*β* =  − 0.39, *p* < 0.001) and lower education (*β* =  − 0.26, *p* = 0.005) were associated with lower life satisfaction (Table 4). Adding the maturity subscales in step 2 significantly improved the model and added another 10.4% of explained variance, *F*(7,82) = 9.09, *p* < 0.001, *R*^2^ = 0.44. Interestingly, growing up slower remained significant (*β* =  − 0.27, *p* = 0.004), whereas education did not (*β* =  − 0.07, *p* = 0.454). Personal maturity approached significance (*β* = 0.26, *p* = 0.061) suggesting a positive association with life satisfaction beyond demographic and medical factors (Table 4).

**Table 4:** Hierarchal linear regression on satisfaction with life

Variable	Life satisfaction		
	*B* (SE)	*β*	*p*-value
Step 1: *R*^2^ = .33, *F*(4,85) = 10.59, *p* < .001			
Relationship status: partnered^a^	0.24 (0.30)	0.08	.420
Education: lower^b^	− 0.72 (0.25)	− 0.26	**.005**
Diagnosis: brain tumor^c^	− 0.48 (0.29)	− 0.16	.106
Growing up slower^d^	− 1.54 (0.36)	− 0.39	**.000**
Step 2: *R*^2^ = .44, *F*(7,82) = 9.09, *p* < .001			
Relationship status: partnered^a^	0.35 (0.32)	0.12	.272
Education: lower^b^	− 0.21 (0.27)	− 0.07	.454
Diagnosis: brain tumor^c^	− 0.08 (0.30)	− 0.03	.787
Growing up slower^d^	− 1.08 (0.36)	− 0.27	**.004**
Financial maturity	0.25 (0.17)	0.18	.146
Social maturity	0.03 (0.19)	0.02	.880
Personal maturity	0.45 (0.24)	0.26	.061

*ΔR*^2^ = .10, *ΔF*(3,82) = 5.07, *p* = .003; reference categories: ^a^vs. single; ^b^vs. high; ^c^vs. non-CNS; ^d^vs. similar/faster

## Discussion

To our knowledge, this is the first study to quantitatively assess different aspects of perceived maturity and examine associations with life satisfaction among young adult survivors of childhood cancer. Results indicate that childhood cancer can have a substantial impact on perceived development, with most survivors reporting they grew up faster than their peers and they felt rather mature. Perceived maturity, and personal maturity in particular, was linked to higher life satisfaction, but certain demographic and medical factors can play a role. Our findings are consistent with studies demonstrating positive growth (i.e., personal maturation/development) as well as high levels of life satisfaction among most, but not all, survivors [7, 8].

Many survivors in this study felt they grew up faster than their peers. Based on previous research, some survivors may see this as a positive difference (e.g., feeling positive about being more mature), while other survivors may experience this negatively (e.g., feeling alienated or not fitting in with peers [7, 9, 13, 32]). Survivors felt most mature about aspects related to financial (i.e., school/job performance, managing bills/insurances) and personal maturity (i.e., managing own health, autonomy), whereas survivors felt least mature about aspects of social maturity (i.e., dating, family planning). Within the financial maturity subscale, survivors rated school job performance highest, although they perceived their financial independence as lowest. These findings are similar to reports from survivors of adolescent and young adult cancer (AYA), who also experience challenges regarding employment and financial stability [33]. Yet, our findings contrast reports of AYA survivors who struggle with education [33]. One explanation may be that treatment coincides with such developmental tasks (pursuing higher education or starting a career) among AYA cancer patients, whereas treatment is typically completed for survivors of childhood cancer before these developmental milestones become salient.

Survivors of brain tumors perceived themselves as least mature and satisfied with life relative to other survivors. This is consistent with research which highlighted diminished social functioning among survivors of pediatric brain tumors [34], as well as psychosocial difficulties across the lifespan [5, 6, 35], potentially resulting in diminished independence. Parents may also become more protective and potentially hamper survivors’ autonomy because of concerns about cognitive functioning, developmental delays, and/or psychosocial difficulties [36, 37]. In contrast, neurotoxic treatment intensity was not related to perceived maturity. The specific mechanisms that may lead to perceptions of decreased maturity/independence among brain tumor survivors but not among those who received high levels of neurotoxic treatments remain unclear.

Partnered survivors perceived themselves as more mature than single survivors, even when controlling for age. This is not surprising given that engaging in more serious relationships/marriage is one developmental task in young adulthood that is often used as an indicator of maturity. At the same time, having a partner may also increase opportunities to accomplish other indicators of maturity, such as financial stability due to a shared income, buying a house, or family planning. Thus, relationship status is intertwined with other aspects of maturity, along with the simple progression of aging, and should be considered when discussing development with survivors. Providers may also address potential difficulties with dating and sexual health if it appears to hamper survivors’ overall quality of life [6, 19–21].

Interestingly, no sex differences in maturity and satisfaction with life were identified in this study. This is in line with previous satisfaction research [24–28, 38], but it is also surprising given that sex differences in quality of life are commonly found. It is also possible that interactions may exist, but our sample size and limited power precluded testing these potential effects. For example, some research has indicated that male brain tumor survivors may have more difficulties with developmental milestones than females [39]. Thus, additional research is needed to further contextualize and measure aspects of quality of life vs. life satisfaction, as well as to examine interactions of demographic and medical factors.

Survivors’ perceptions of growing up slower than peers and lower education were associated with lower life satisfaction. Yet, the number of survivors falling into either subgroup was small, which is promising. Our findings highlight overall high satisfaction with life, which is consistent with the research of positive growth following cancer, aiding in a successful transition to adulthood and attainment of developmental milestones [40, 41]. This finding also corroborates previous research in congenital heart disease/heart transplant survivors that found maturity is associated with increased quality of life [42, 43]. This suggests that emotional and personal stability/growth, as well as autonomy could positively contribute to feelings of responsibility and control over life, and therefore reinforce survivors’ life satisfaction.

### Study limitations

This study presents novel insights, but some limitations should be considered. First, recruitment of a healthy control group would facilitate direct comparisons regarding development and maturity. Second, although our sample was sizeable and analyses had acceptable power, more detailed analyses were not possible. Interactions between certain demographic and medical factors should be tested (e.g., male vs. female brain tumor survivors vs. others), as well as effects among more refined groups regarding age at diagnosis (e.g., younger vs. older adolescents). Third, perceptions of maturity were self-reported and may differ from how others perceive survivors’ maturity. Yet, such self-perceptions may be more salient indicators of satisfaction than the attainment of normative developmental milestones in itself, which needs further investigation. Fourth, the family of origin might play a crucial role in the maturation of survivors and reaching their autonomy, but this was outside the scope of this study. Finally, and as indicated above, this study used cross-sectional data, limiting our ability to infer causal relationships.

### Clinical implications

To maximize satisfaction with life throughout survivorship, healthcare providers should regard maturation and development as a process across the life span and support childhood cancer survivors in accomplishing developmental tasks in each life phase. Specific attention may be needed for survivors who feel their development was slowed by their cancer experience, along with survivors of brain tumors. Healthcare providers are advised to screen survivors for potential delays or burdens related to maturity and development, for which the items of the Maturity Scale may be useful. Moreover, psychosocial providers should counsel survivors regarding dating/romantic relationships, financial independence, family planning, and emotional stability in a developmentally appropriate way. A specific focus on promoting positive gains of personal maturity (emotional stability, goal setting) may be warranted, as this could increase overall satisfaction with life among all survivors of childhood cancer.

## Data Availability

De-identified data supporting the findings of this study are available from the corresponding author upon request.
